# Aerobic Exercise Response Variation and Cardiorespiratory Fitness in Adults with Coronary Heart Disease: An SDir Meta-Analysis of Randomized Controlled Trials

**DOI:** 10.3390/jcdd13070307

**Published:** 2026-07-03

**Authors:** George A. Kelley, Kristi S. Kelley, Brian L. Stauffer

**Affiliations:** 1 School of Public and Population Health, Boise State University, Boise, ID 83725, USA; kristikelley@boisestate.edu; 2 School of Kinesiology, Boise State University, Boise, ID 83725, USA; 3 Department of Medicine, Division of Cardiology, Denver Health Medical Center, Denver, CO 80204, USA; brian.stauffer@cuanschutz.edu; 4 Department of Medicine, Division of Cardiology, University of Colorado School of Medicine, Aurora, CO 80045, USA

**Keywords:** exercise, aerobic, precision exercise, response variation, heart disease, cardiorespiratory fitness, meta-analysis

## Abstract

Background: Given that true exercise response variation on cardiorespiratory fitness in adults with coronary heart disease (CHD) is not known, this study addressed this gap. Methods: Randomized controlled trials (RCTs) comparing continuous aerobic exercise (CAE) to controls in adults ≥18 years of age with CHD were included. The primary outcome was exercise-associated inter-individual response differences (IIRDs) in cardiorespiratory fitness (VO_2peak_ in ml·kg^−1^·min^−1^). Using the inverse variance heterogeneity (IVhet) model, a standard deviation of individual response difference (SD_ir_) meta-analysis was conducted. Ninety-five percent confidence intervals (CIs) and prediction intervals (PIs) were calculated. Results: Twenty-eight RCTs representing 1383 participants (725 CAE, 658 control) were included. Statistically significant and clinically important improvements (≥1.0 mL·kg^−1^·min^−1^) were observed for VO_2peak_ as a result of CAE ($\bar{X}$, 3.6, 95% CI, 2.8 to 4.4 mL·kg^−1^·min^−1^, *p* < 0.001), but no statistically significant or clinically important IIRD based on the SD_ir_ were found ($\bar{X}$, 0.9, 95% CI, −1.5 to 2.0 mL·kg^−1^·min^−1^; 95% PI, −2.4 to 2.7). Based on GRADE, the strength of evidence was of low certainty. Conclusions: There is low certainty evidence that CAE results in statistically significant and clinically important improvements in VO_2peak_ in adults with CHD, but no exercise-associated IIRD was observed once properly accounted for.

## 1. Introduction

Coronary heart disease (CHD), a subset of cardiovascular disease, is a major public health problem worldwide, with an estimated 315 million people affected in 2022 [1]. In the United States (US), the number of adults 20 years of age and older with CHD was estimated to be 20.5 million in 2020 [2]. Not surprisingly, the worldwide economic costs associated with CHD are varied but substantial, with most countries well-exceeding the total health expenditure per capita, with a pooled percentage of 21.7% of the gross domestic product across all included countries [3]. In the US, the total costs of CHD were estimated to be $260 billion (USD) in the year 2020, with an expected increase to $584 billion (USD) by 2050 [4]. Most notably, CHD is the leading cause of mortality worldwide (9.1 million deaths in 2021) [5], as well as in the US (371,506 deaths in 2022) [2].

A previous systematic review with meta-analysis of randomized controlled trials (RCTs) reported that higher cardiorespiratory fitness, a clinical vital sign [6], was associated with a pooled hazard ratio reduction of 68% for all-cause mortality among those with coronary heart disease (CHD) [7]. Aerobic exercise, a highly recommended lifestyle modification for those with CHD [2,8], has been shown to increase cardiorespiratory fitness, assessed as VO_2peak_ in ml·kg^−1^·min^−1^, in adults with CHD. For example, in a recent overview of previous meta-analyses in which the authors conducted their own meta-analysis, continuous aerobic exercise (CAE) resulted in statistically significant and clinically important increases of 3.8 mL·kg^−1^·min^−1^ (95% CI, 3.2, 4.4 mL·kg^−1^·min^−1^) in VO_2peak_ among adults with CHD [9]. Irrespective of population, it is generally believed that the primary mechanism driving this exercise-induced increase is increased blood flow and oxygen delivery [10].

Precision exercise medicine, an outgrowth of precision medicine, is based on the notion that people respond to exercise differently and thus, there is a need for individualized exercise prescriptions to maximize benefits for the outcome(s) of interest [11,12,13,14,15,16,17,18]. However, the basis for true exercise-response variation, also known as interindividual response differences (IIRD), has historically not been properly assessed by first examining for random measurement error and within-subject variability, i.e., physiological responses as a result of changes exclusive of the exercise intervention (physical activity beyond the exercise intervention, diet, environmental and behavioral factors) [19,20,21]. As a result, advancement to examine for potential moderators and mediators, dose–response associations, and genetic interactions, cannot be justified, i.e., false exercise-associated IIRD [20]. The negative result of advancing to such without proper assessment could lead to misguided effort and resources examining for exercise-associated IIRD as well as needless individualized exercise prescriptions [20]. In addition, it may exacerbate healthcare inequalities [22]. Thus, excluding factors such as personal preferences for modality (walking, cycling, etc.), general exercise recommendations such as those from the American Heart Association and the American Association of Cardiovascular and Pulmonary Rehabilitation in those with CHD may suffice [23].

To illustrate the issue of the lack of exercise-associated IIRD specific to cardiorespiratory fitness, a detailed review by Williamson et al., with a focus on changes in VO_2max_ in ml·kg^−1^·min^−1^, concluded that when appropriately assessed, exercise-associated IIRD in VO_2max_ were not larger than random within-subject variation [24]. In their re-analysis of data from the HEalth, RIsk factors, exercise Training And GEnetics (HERITAGE) Family Study in which random within-subject variation was properly accounted for by using the standard deviation of individual response (SD_ir_) approach, the change in VO_2max_ was actually larger in the control (±5.6 mL·kg^−1^·min^−1^) versus exercise group (±3.7 mL·kg^−1^·min^−1^) for the one study that included a control group [24]. These findings suggest that there is probably no need to move forward and examine for exercise-associated IIRD despite previous claims that genetic factors determine more than 50% of the individual differences in aerobic capacity [25].

Currently, the preferred method for examining exercise-associated IIRD is the SD_ir_ approach [19,20,26] via meta-analysis given the increased power of the latter over individual RCTs [27]. First proposed in the exercise field by Hopkins and described in more detail in the methods section of this paper, the SD_ir_ approach in an RCT treats the change outcome standard deviations in the exercise and control groups as point estimates, estimates the variance for each, and then calculates the difference between the two [19]. If the SD_ir_ is significant and in the direction of improvement for the exercise versus control group, one may then conclude that exercise-associated IIRD occur and then proceed to examine for potential moderators, mediators, dose–response effects, as well as genetic interactions regarding exercise-associated IIRD. However, if no significant SD_ir_ improvement in favor of the exercise group is found, there is probably no need to examine for any exercise-associated IIRD.

The lack of exercise-associated IIRD has consistently been reported in multiple studies and across a variety of outcomes, populations, and risk factors. In addition to the work of Williamson et al. [24], a lack of exercise-associated IIRD based on SD_ir_ meta-analyses of RCTs have consistently been found for changes in cardiorespiratory fitness as a result of (1) exercise-based cardiac rehabilitation in heart transplant patients [28], (2) resistance training in older adults [29], and (3) exercise training in adults with different cardiovascular disease risk factors [30]. Similar results using SD_ir_ meta-analyses of RCTs have been found for other outcomes that include: (1) changes in adiposity among children and adolescents with overweight and obesity as a result of aerobic exercise [31,32], (2) changes in resting systolic and diastolic blood pressure as a result of walking [33], qigong [34], and tai chi [35] in adults, (3) exercise and changes in body weight in adults [36], (4) exercise, waist circumference and body mass index in adults [30], and (5) the effects of exercise among adults with anxiety and stress-related disorders [37]. One exception is a recent meta-analysis by Meyler et al. in which changes in VO_2max_ from controlled studies was greater when prescribing exercise intensity based on physiological thresholds versus traditional anchors [38]. Finally, a recent systematic review that included previous meta-analyses that examined IIRD concluded that there is insufficient evidence to support true IIRD in physiological adaptations as a result of aerobic exercise training interventions [39].

To the best of the investigative team’s knowledge, no previous SD_ir_ meta-analysis has been conducted on the effects of aerobic exercise training on cardiorespiratory fitness, assessed as VO_2peak_ in ml·kg^−1^·min^−1^, in adults with CHD. The determination of such is important from a research perspective to avoid unneeded follow-up analyses and additional studies, and from a practical perspective, the development of recommendations for exercise prescription based on sound science. Leveraging data from the recent umbrella review and meta-analysis of Gomes-Neto et al. [9], this gap is addressed. The working hypothesis is the null, that is, no statistically or clinically significant aerobic exercise-associated IIRD will be found in VO_2peak_ once properly accounted for using the SD_ir_ approach.

## 2. Materials and Methods

### 2.1. Overview

Where appropriate, the 2020 reporting guidelines from the Preferred Reporting Items for Systematic Reviews and Meta-Analyses (PRISMA) statement were followed (Table S1) [40]. The protocol for this study was registered on 5 June, 2025 in Open Science Framework (https://osf.io/txrqn/registrations) but not published in a peer-reviewed journal. Post-hoc changes to the a priori protocol, including reasons, appear throughout the methods that follow.

### 2.2. Data Source and Study Selection

Selected data was derived from a recent umbrella review [9] that included a meta-analysis of individual RCTs on the effects of continuous aerobic exercise (CAE) training on aerobic capacity, reported as VO_2peak_ in ml·kg^−1^·min^−1^, that included 30 RCTs representing 1453 men and women (752, CAE, 701 control) ≥18 years of age with CHD [41,42,43,44,45,46,47,48,49,50,51,52,53,54,55,56,57,58,59,60,61,62,63,64,65,66,67,68,69,70]. Separate meta-analyses on high-intensity interval training versus control, resistance training versus control, and combined resistance training versus control, were not included because of the small number of studies for each (≤6) [9]. In addition, meta-analyses on high-intensity interval training versus moderate-intensity continuous training as well as combined CAE and resistance training versus moderate-intensity continuous training were not included because the focus of the current study is on an SD_ir_ meta-analysis that requires a control group to address the research team’s purpose. Based on the decision framework of Garner et al. on when to update a systematic review [71], it was decided by the investigative team that no such update was needed. In addition, it has been suggested that investigators leverage data from previously published meta-analyses for conducting IIRD meta-analyses [72].

Given recent research showing that retracted studies included in a meta-analysis can negatively impact the results and interpretation of the meta-analysis [73], the first author used the retracted study filter in PubMed on 6 June 2025 to examine each initially included study for potential retraction. If retracted, it was to be excluded from all analyses. The post-hoc decision to search PubMed versus Web of Science was based on the fact that not all studies were indexed in Web of Science.

### 2.3. Data Abstraction

Using Microsoft^®^ Excel^®^ for Microsoft 365 (version 2605), a codebook was developed and pilot tested by the first two authors with input from the third author. Independent, dual data extraction from the previous meta-analysis was conducted by the first two authors using separate Excel codebooks. They then met and reviewed their selections for agreement. Any disagreements were resolved through mutual agreement or via adjudication by the third author if agreement could not be reached. During the data abstraction process, a post-hoc decision was made to abstract data from the original 30 studies and recalculate all effect sizes because the data reported in the original meta-analysis did not include decimal places for the primary outcome of interest, i.e., VO_2peak_ in ml·kg^−1^·min^−1^ [9]. This was accomplished using the same procedure as for our original data abstraction and included study, subject, intervention, outcome and characteristics (VO_2peak_). During the data abstraction process, another post-hoc decision was made to delete two studies because they included the same participants as two other included studies [60,64]. Using Gwet’s AC1 statistic [74], overall agreement for data abstraction prior to correcting disagreements was 0.99 (95% CI, 0.994, 0.999).

### 2.4. Research Synthesis

#### 2.4.1. Treatment Effect Meta-Analysis

Using the original metric, a traditional treatment effects meta-analysis, i.e., change outcome difference in the CAE group minus the change outcome difference in the control group, was conducted to determine the effects of CAE on the primary outcome, VO_2peak_ in ml·kg^−1^·min^−1^. To maintain independence, studies that included multiple CAE groups were pooled into one overall effect size. If both intention-to-treat and per-protocol analyses were performed, results from the more conservative intention-to-treat approach were used. Given that only two of the original 30 studies provided change outcome standard deviations [55,58], these were estimated for the other studies according to the formula of Follman et al. [75]. The correlation used in this formula was derived from the one study that provided the necessary data to calculate such, resulting in a correlation coefficient of 0.86 for the CAE groups and 0.90 for the control groups [58]. Results from each study were then pooled using the inverse-variance heterogeneity (IVhet) model, not to be confused with inverse-variance weighting [76,77,78,79]. This model was chosen over the many others [80], including the random-effects model used in the original meta-analysis [9], because the IVhet model has been shown to provide better coverage probabilities for 95% confidence intervals (CI) than other models, including random-effects models [76,77,78,79]. Two-tailed z-alpha values ≤0.05 and 95% CI that do not include the null (0) were considered statistically significant. In addition, the magnitude of treatment effect changes in VO_2peak_ was compared to a minimal clinically important difference (MCID) of 1.0 mL·kg^−1^·min^−1^ given previous research demonstrating a 9% decrease in the relative risk for all-cause mortality [81]. Based on previous suggestions, the following probabilistic anchors were used to interpret results: <0.5%, most unlikely or almost certainly not, 0.5–5%, very unlikely, 5–25%, unlikely or probably not, 25–75%, possibly, 75–95%, likely or probably, 95–99.5%, very likely, >99.5%, most likely or almost certainly [82].

*Statistical heterogeneity* was examined using the Q statistic, with an alpha value ≤0.10 considered to represent statistically significant heterogeneity [83]. Inconsistency was examined using the *I*^2^ statistic, including 95% CI [84]. In addition, 95% prediction intervals (PI), a better estimator than Q and *I*^2^ for between-study heterogeneity and inconsistency, were also calculated [85]. Ninety-five percent PI include an absolute measure ($\tau^{2}$) of between-study heterogeneity in their calculation and are a better estimator than Q and *I*^2^ for between-study heterogeneity and inconsistency [85]. While 95% CI are an index of precision, telling us how precisely one has estimated a pooled effect, 95% PI are a measure of dispersion, telling one how widely the pooled effect size varies. Thus, 95% PI estimates what result an investigator might find for their outcome of interest if they conducted a new study from the same population included in the meta-analysis.

*Small-study effects* (publication bias, etc.) were examined visually using the Doi plot and quantitatively using the Luis Furuya-Kanamori (LFK) index, both have which have been reported to be superior to the funnel plot and Egger’s regression intercept test [86]. Values for the LFK index within ±1 suggest no asymmetry, ±1 but within ±2 minor asymmetry, and values exceeding ±2 major asymmetry [86].

*Leave-one-out analysis* was conducted to examine the influence of each study on the overall results. In addition, *outlier analysis* was conducted by deleting those studies in which their pooled estimates and 95% CI were outside the overall pooled estimate and 95% CI.

*Risk of bias and the strength/certainty of evidence* was not assessed because it was previously reported in the umbrella meta-analysis for which data for the current IIRD meta-analysis is derived [9]. Using the Grading of Recommendations Assessment, Development and Evaluation (GRADE) instrument, RCTs of CAE were considered to provide low-certainty evidence that was downgraded because of risk of bias and inconsistency [9].

#### 2.4.2. SD_ir_ Meta-Analysis

For the primary purpose of this study, IIRD for changes in VO_2peak_ in ml·kg^−1^·min^−1^ were assessed using the recommended SD_ir_ approach [19,20,26]. This was calculated by treating CAE and control group standard deviations as point estimates, calculated from each study as follows [19]:

$$\sqrt{{SD}_{cae}^{2}-\;{SD}_{c}^{2}}$$

whereby ${SD}_{cae}^{2}$ represents the standard deviation for the CAE group and ${SD}_{c}^{2}$ represents the standard deviation for the control group. The standard error for these point estimates was calculated using the following formula [19]:

$$SE=\sqrt{2\left(\frac{{SD}_{cae}^{4}}{{DF}_{cae}}+\;\frac{{SD}_{c}^{4}}{{DF}_{c}}\right)}$$

where DF represents the degrees of freedom minus 1.

Positive values based on the SD_ir_ approach suggest greater IIRD, i.e., variability, for changes in VO_2peak_ in the CAE versus control groups, while negative values suggest the opposite. The SD_ir_ results from each study were then pooled using the same methods as for the traditional treatment effects meta-analysis previously described. Pooled SD point estimates and 95% CI were calculated from the square root of each value [36]. For lower 95% CI that were negative, the sign was first ignored, the square root calculated, and the sign then reapplied. Tau (τ) was used as an absolute measure of between-study heterogeneity. Because of missing data for different variables from different studies, no subgroup, moderator, mediator, or meta-regression analyses were conducted. In addition, because such analyses are considered observational in an aggregate data meta-analysis, given that studies are not randomly assigned to covariates, they do not support causal inferences, and thus, are considered exploratory, i.e., hypothesis generating [87].

Statistical analyses were conducted using the user-written metan (version 4.08.1) and KAPPAETC (version 1.0) routines in Stata (version 16.1), Stata itself (version 16.1), and the Excel add-ins Meta XL (version 5.3) and SSC-Stat (version 3.0).

## 3. Results

Overall study, participant and intervention characteristics are shown in Table 1, while study characteristics, as well as participant and intervention characteristics at the study level, are shown in Table S2.

### 3.1. Study Characteristics

The 28 included RCTs represented a total of 1383 participants (725 CAE, 658 control), ranging from 8 to 59 in the CAE groups ($\bar{X}$ ± SD, 26 ± 13, Mdn = 23) and 8 to 59 in the control groups ($\bar{X}$ ± SD, 23 ± 12, Mdn = 19) [41,42,43,44,45,46,47,48,49,50,51,52,53,54,55,56,57,58,59,61,62,63,65,66,67,68,69,70]. None of the studies were listed as being retracted. Only four studies (14.3%) provided a Consolidated Standards of Reporting Trials (CONSORT flow diagram [45,46,58,61], while only four others provided a priori power estimates [45,54,61,62]. Studies were conducted in 14 different countries, [41,42,43,44,45,46,47,48,49,50,51,52,53,54,55,56,57,58,59,61,62,63,65,66,67,68,69,70] with the majority of participants (89.3%) consisting of men for the 24 studies (85.7%) in which data were available [42,43,44,45,46,47,48,49,50,51,52,53,54,55,56,57,58,59,61,62,65,66,67,68]. Dropouts calculated from 24 studies (85.7%) ranged from 0 to 23% in the CAE groups ($\bar{X}$ ± SD, 4.9 ± 7.2, Mdn = 0) versus 0 to 36.8% in controls ($\bar{X}$ ± SD, 5.6 ± 9.5, Mdn = 0) [41,42,43,44,45,46,48,49,50,51,57,58,59,62]. Two studies (7.1%) reported adverse events [58,59]. One reported two adverse events, one because of an embolism of air during cardiac catheterization that was successfully treated by direct aspiration of air from the right coronary artery and another patient because of progression of coronary artery disease with an increase in angina during the third week of the study [58]. A second study reported no adverse events during exercise testing or training during the initial two months but during the subsequent 10 months, two patients in the control group were hospitalized for decompensation of heart failure, both of which were stabilized after a brief uncomplicated hospital stay. Another patient in the exercise group had sudden cardiac death 9 months after beginning the study [59]. Nine studies (32.1%) reported funding from various sources for their research [41,43,45,52,54,59,62,65,70].

### 3.2. Participant Characteristics

Table 1 provides a summary of study, particpant, and intervention characteristics. For the 26 studies (92.8%) that reported data for the CAE and/or control groups [41,42,43,45,46,47,48,49,50,51,52,53,54,55,56,57,58,59,60,61,62,63,64,65,66,67,68,69], mean group ages from the studies ranged from 51.5 to 72.2 years in the CAE groups ($\bar{X}$ ± SD, 59.4 ± 6.0, Mdn = 58.9) and 52.0 to 72.9 in the control groups ($\bar{X}$ ± SD, 60.3 ± 5.6, Mdn = 59.5). Baseline mean group body mass index (BMI) values from 14 studies (50%) ranged from 23.7 to 29.9 kg·m^2^ in the CAE groups ($\bar{X}$ ± SD, 27.4 ± 4.1 kg·m^2^, Mdn = 26.9) to 23.7 to 30.0 kg·m^2^ in the control groups $(\bar{X}$ ± SD, 27.5 ± 4.0 kg·m^2^, Mdn = 27.0) [41,42,44,45,46,49,50,54,58,59,62,65,66,68]. For the 27 studies (96.4%) in which baseline data were available [41,42,43,44,45,46,47,48,49,50,51,52,53,54,56,57,58,59,61,62,63,65,66,67,68,69,70], values for VO_2peak_ ranged from 12.6 to 32.0 mL·kg·min^−1^ in the CAE groups ($\bar{X}$ ± SD, 20.2 ± 6.4, Mdn = 19.9) and 11.7 to 32.6 mL·kg^−1^·min^−1^ in the control groups ($\bar{X}$ ± SD, 20.3 ± 6.4, Mdn = 20.0). Assessment of VO_2peak_ (27 studies, 96.4%) [41,43,44,45,46,47,48,49,50,51,52,53,54,55,56,57,58,59,60,61,62,63,64,65,66,67,68,69,70] was conducted using either cycle ergometry (14 studies, 50%) or treadmill (13 studies, 46.4%) exercise.

Risk factors for some participants included type 2 diabetes, hypercholesterolemia, hypertension, cigarette smoking, and overweight/obesity. Medications taken by some participants included aspirin, cholesterol-lowering drugs (statins, etc.), diuretics, beta-blockers, nitrates, angiotensin converting enzyme inhibitors, calcium antagonists, anti-coagulants, and anti-hypertensive drugs.

### 3.3. Intervention Characteristics

Length of CAE training, in weeks, ranged from 2 to 28 ($\bar{X}$ ± SD, 12.5 ± 8.0, Mdn = 12) [41,42,43,44,45,46,47,48,49,50,51,52,53,54,55,56,57,58,59,61,62,63,65,66,67,68,69,70]. When reported in terms of times per week (24 studies, 85.7%), frequency ranged from 1 to 12 days ($\bar{X}$ ± SD, 3.6 ± 1.9, Mdn = 3.0) [41,42,43,44,45,46,47,48,49,50,51,52,54,55,56,57,59,61,62,63,65,66,68,69,70]. Three other studies reported frequency in terms of times per day: 6 [53], 6 to 8 [58], 2 [67]. Duration of training ranged from 20 to 60 min per session ($\bar{X}$ ± SD, 31.0 ± 8.9, Mdn = 30) [41,42,43,44,45,46,47,48,49,50,51,52,53,54,55,56,57,58,59,61,62,63,65,66,67,68,69,70]. Intensity of training was reported using a variety of methods (VO_2peak_, maximum heart rate, maximum heart rate reserve, anaerobic threshold, individual angina-free exercise threshold, Borg scale) [41,42,43,44,45,46,47,48,49,50,51,52,53,54,55,56,57,58,59,61,62,63,65,66,67,68,69,70]. Based on American College of Sports Medicine categories [88], intensity, collectively, was considered to be moderate to vigorous. Training modality (26 studies, 92.9%) [41,42,43,44,45,46,47,48,49,50,51,52,53,54,55,56,57,59,60,61,63,64,65,66,67,68,69,70] varied but most commonly included cycle ergometry and/or treadmill exercise (walking, jogging, running). All studies appeared to include a supervised exercise group [41,42,43,44,45,46,47,48,49,50,51,52,53,54,55,56,57,58,59,61,62,63,65,66,67,68,69,70], with one including both supervised and unsupervised exercise in the same group [63] and another including separate supervised and unsupervised exercise groups [68]. Compliance (12 studies, 42.9%) [41,43,45,46,47,48,49,50,57,58,61,62], defined as the percentage of exercise sessions attended, ranged from 75 to 100% ($\bar{X}$ ± SD, 87.5 ± 7.6, Mdn = 89.8).

### 3.4. Treatment Effect Results

**Figure 1:**
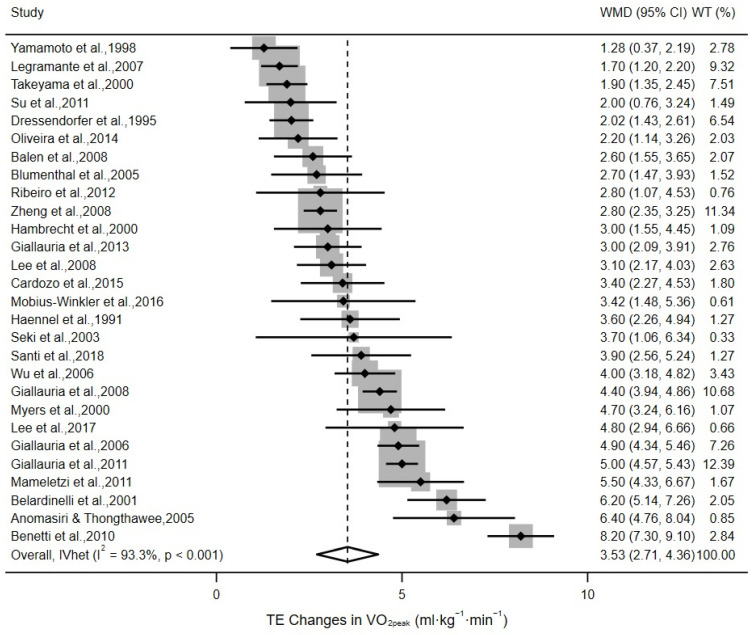
Treatment effect changes in VO_2peak_ in ml·kg^−1^·min^−1^ from 28 studies [41,42,43,44,45,46,47,48,49,50,51,52,53,54,55,56,57,58,59,61,62,63,65,66,67,68,69,70].

Overall treatment effect results are shown in Table 2, while study-level results are shown in Figure 1. Statistically significant increases in VO_2peak_ (z = 8.8, *p* <0.001) were observed, equivalent to a relative increase of 18.8% (95% CI, 14.4% to 23.2%). Statistically significant heterogeneity (Q = 231.7, *p* <0.001) and large inconsistency (*I*^2^ = 88.3%, 95% CI, 72.2% to 93.6%) were observed. However, the 95% PI did not include the null (0). Using suggested probabilistic anchors, the probability of exceeding an MCID in VO_2peak_ of 1.0 mL·kg^−1^·min^−1^ was 93.6% (likely or probably clinically important). Minor asymmetry suggestive of small study effects was observed (Figure S1).

With each study deleted from the model once, results remained statistically significant across all deletions, ranging from 3.4 (95% CI, 2.6 to 4.2) to 3.8 (95% CI, 3.0 to 4.6) ml·kg^−1^·min^−1^ (Figure S2). With six outlier studies deleted [43,44,49,56,67,69], results remained statistically significant, equivalent to a relative increase in VO_2peak_ of 18.7% (95% CI, 15.6% to 21.9%) (Table 2, Figure S3). Similar to our overall findings, statistically significant heterogeneity (Q = 70.3, *p* < 0.001) and a large amount of inconsistency (I^2^ = 70.1%, 95% CI, 23.9% to 84.2%) were observed. The 95% PI did not include the null (0) and was narrower compared to results when all studies were included. The probability of exceeding a MCID in VO_2peak_ of 1.0 mL·kg^−1^·min^−1^ was 99.2% (very likely to be clinically important). No asymmetry suggestive of small-study effects was found (Figure S4).

### 3.5. SDir Results

For the primary purpose of this study, the overall results for the SDir meta-analysis can be found in Table 2. As can be seen, the 95% CI included the null for all studies, as well as when two outlier studies were deleted from the model, suggesting no IIRD. Congruent with these findings, the 95% PI was wide. The probability of clinically important IIRD was 56.8% (only possibly clinically important). With two outlier studies deleted [43,61], results were similar (56.2%, only possibly clinically important).

## 4. Discussion

### 4.1. Overall Findings

The overall findings for the primary purpose of the current study suggest that neither statistically significant nor clinically important exercise-associated IIRD occur in VO_2peak_ as a result of CAE in adults with CHD when properly assessed using the SD_ir_ approach. Rather, such differences most likely derive from random (measurement error, day-to-day biological variation) and/or within-subject variation, i.e., changes in behavior (diet, etc.) and/or environment [26]. With the exception of one study [38], these findings are consistent with all others as they pertain to exercise-associated IIRD and VO_2peak_ and VO_2max_ in other populations [28,29,30]. The current findings, as well as those from other studies [28,29,30], challenge the long-held notion that as much as 50% of the response to changes in maximum oxygen consumption as a result of CAE is heritable [25,89].

While not the primary purpose of the current study, the results demonstrated both statistically significant and clinically important treatment effect increases in VO_2peak_ as a result of CAE. These findings were consistent when each study was deleted from the model once, as well as when six outliers were deleted [43,44,49,56,67,69]. In addition, a lack of small-study effects (publication bias, etc.) was observed. These treatment effect results are consistent with those of the umbrella review of Gome-Neto et al. [9], and are important given that increases in VO_2peak_ after cardiac rehabilitation have been shown to predict long-term survival in patients with CHD [90].

To summarize, CAE resulted in clinically important improvements in VO_2peak_ but no exercise-associated IIRD. The implications for this as it pertains to research and practice are described in the sections that follow.

### 4.2. Implications for Research

From the investigative team’s perspective, there are several implications for future research. First, based on the current findings, there appears to be no need to examine for potential moderators, mediators, or genetic interactions as they pertain to the effects of CAE training on changes in VO_2peak_ in adults with CHD. Doing so may result in misdirected effort and resources [20]. Second, it is suggested that future RCTs properly assess exercise-associated IIRD before advancing to explore for potential moderators, mediators, and genetic interactions as they pertain to changes in VO_2peak_ in adults with CHD. Not doing so could lead to unnecessary time and resources, as well as inappropriate conclusions [20]. Guidelines for the assessment of such are provided elsewhere [20,91]. Second, it is suggested that future meta-analyses, both prospective and retrospective, also properly assess for IIRD [72]. Third, there is a need to properly assess for potential exercise-associated IIRD for other outcomes such as resting systolic and diastolic blood pressure, lipids and lipoproteins, glycated hemoglobin, and left ventricular ejection fraction, as well as the effects of different types of exercise training (high-intensity interval training, resistance training, combined training) on both VO_2peak_ as well as the aforementioned outcomes in patients with CHD. Fourth, while beyond the scope of the current study, future research should focus on the development of valid and reliable methods for assessing which random and within-subject variation factors are associated with changes in VO_2peak_ as well as other outcomes, in adults with CHD. Fifth, from a reporting perspective, future RCTs and meta-analyses should report complete data (change outcome sample sizes, means, standard deviations, etc.) in both exercise and control groups so that the proper assessment of IIRD can be accomplished using the preferred SD_ir_ approach [19,20,26]. For example, in the current meta-analysis, only two studies provided change outcome SDs for VO_2peak_ [55,58], resulting in the need to estimate these for the other studies.

In summary, there is a need for improved research on the assessment and reporting of exercise-associated IIRD on VO_2peak_ as well as other variables in adults with CHD.

### 4.3. Implications for Practice

The current findings reinforce the importance of CAE for increasing VO_2peak_ in adults with CHD. Thus, CAE should continue to serve as a cornerstone of cardiac rehabilitation programs. However, the results do not support the use of precision exercise medicine for increasing VO_2peak._ Given the former, it is suggested that practitioners adhere to the general exercise guidelines recommended in the recent 2024 core components of cardiac rehabilitation programs statement from the American Heart Association and American Association of Cardiovascular and Pulmonary Rehabilitation [23]. However, while the precision exercise approach cannot be recommended at this time, it should not be interpreted within the context of dismissing patient preferences. For example, to increase adherence, practitioners should allow CHD patients to select the type of CAE (walking, cycling, swimming, etc.) they would like to take part in. In the current meta-analysis, cycle ergometry exercise was the primary modality followed by walking and/or jogging, primarily on a treadmill, Additional information regarding key factors associated with adherence to physical exercise in patients with chronic diseases, as well as in older adults, has been described in detail elsewhere [92].

To summarize, adherence to general guidelines for exercise-based cardiac rehabilitation may suffice for increasing VO_2peak_ in adults with CHD.

### 4.4. Strengths and Potential Limitations

From the investigative team’s viewpoint, the major strength of this study is that it is the first, to the best of the authors’ knowledge, to use the SD_ir_ approach to properly examine for potential IIRD on VO_2peak_ as a result of CAE in adults with CHD. These findings are important as they suggest that an examination for potential moderators, mediators, and genetic interactions is not necessary.

In addition to the major strength, there are several potential limitations. First, the overall strength of the data for CAE based on GRADE and as reported by Gomes-Neto et al. was considered to provide low-certainty evidence that was downgraded because of risk of bias and inconsistency [9]. The current authors agree with this assessment. Second, the need to estimate SDs for all but two studies in the current IIRD meta-analysis introduces the potential for error in the actual SDs [53,56]. Third, while all studies were RCTs, the SD_ir_ approach assumes that the combined effect of random and within-subject variation is equal between the intervention and control groups [26]. However, it is still the preferred method for examining exercise-associated response heterogeneity [26]. Fourth, since this was an aggregate data meta-analysis, the potential for ecological fallacy exists [93]. Finally, these findings should be interpreted while considering the temporal heterogeneity of the studies given the gap in scientific, technological, methodological, and application knowledge between the years of the included studies (1995 to 2018).

## 5. Conclusions

The current findings suggest that while CAE results in statistically significant and clinically important improvements in VO2peak in adults with CHD, there is no exercise-associated IIRD once properly accounted for. The lack of exercise-associated IIRD suggests that other random and/or within-subject variation factors are responsible for any differences observed. However, the strength of evidence was of low certainty, suggesting that future well-designed RCTs that report complete data are needed before any final consensus can be reached.

## 6. Patents

Not applicable. There were no patents that resulted from this work.

## Figures and Tables

**Table 1:** Study, participant, and intervention characteristics.

Variable	CAE				Control			
	N	$\bar{X}$ ± SD	Mdn	Min–Max	N	$\bar{X}$ ± SD	Mdn	Min–Max
Participants	28	26 ± 13	23	8–59	28	23 ± 12	19	8–59
Study Dropouts (%)	24	4.9 ± 7.2	0	0–23	24	5.6 ± 9.5	0	0–37
Age (years)	26	59.4 ± 6.0	59	51–72	26	60.3 ± 5.6	59	52–73
BMI (kg·m^−2^)	14	27.4 ± 4.1	27	24–30	14	27.5 ± 4.0	27	24–30
VO_2peak_ (ml·kg^−1^·min^−1^)	27	20.2 ± 6.4	20	13–32	27	20.3 ± 6.4	20	12–33
Length (wks)	28	12.5 ± 8.0	12	2–28	na	na	na	na
Frequency (times/wk)	24	3.6 ± 1.9	3	1–12	na	na	na	na
Duration (min/session)	24	31.0 ± 8.9	30	20–60	na	na	na	na
Compliance (%)	12	87.5 ± 7.6	90	75–100	na	na	na	na

Notes: CAE, continuous aerobic exercise; N, number of studies in which data were available; $\bar{X}$ ± SD, mean ± standard deviation; Mdn, Median; Min–Max, minimum and maximum values; BMI, body mass index; VO_2peak_ ml·kg^−1^·min^−1^, relative peak maximum oxygen consumption; wks, weeks; min, minutes; Compliance (%), percentage of exercise sesssions attended; na, not applicable.

**Table 2:** Treatment effect and IIRD results for VO_2peak_ in ml·kg^−1^·min^−1^.

Variable	Studies (#)	Participants (#)	$\bar{X}$ (95% CI) ^c^	95% PI ^d^
TE ^a^				
- All	28	1383	3.6 (2.8, 4.4) *	0.5, 6.8 *
- Outliers Deleted (n = 6)	22	952	3.6 (2.9, 4.2) *	1.5, 5.6 *
SD_IR_^b^				
- All	28	1383	0.9 (−1.5, 2.0)	−2.4, 2.7
- Outliers Deleted (n = 2)	26	1173	0.8 (−1.4,1.8)	−2.2, 2.5

Notes: ^a^ TE, treatment effects; ^b^ SD_IR_, standard deviation of individual response differences; ^c^ $\bar{X}$ (95% CI), mean and 95% confidence interval; ^d^ 95% PI, 95% prediction interval; *, nonoverlapping 95% confidence or prediction intervals.

## Data Availability

All data are available from the corresponding author upon reasonable request.

## Supplementary Materials

The following supporting information can be downloaded at: https://www.mdpi.com/article/10.3390/jcdd13070307/s1, Table S1: PRISMA Checklist; Table S2: Study, participant, and intervention characteristics of included studies; Figure S1: Doi plot for small-study effects (publication bias, etc.) for changes in VO_2peak_ (all studies); Figure S2: Forest plot for leave-one-out analysis based on changes in VO_2peak_; Figure S3: Forest plot for changes in VO_2peak_ with six outliers deleted from the model; Figure S4: Doi plot for small-study effects (publication bias, etc.) for changes in VO_2peak_ with six outliers deleted from the model.

## Abbreviations

The following abbreviations are used in this manuscript:

CHD	coronary heart disease
CAE	continuous aerobic exercise
IIRD	inter-individual response differences
VO_2peak_	peak oxygen consumption in ml·kg^−1^·min^−1^
IVhet	inverse variance heterogeneity model
SD_ir_	standard deviation of individual responses
CI	confidence interval
CIs	confidence intervals
PI	prediction interval
PIs	prediction intervals
GRADE	Grading of Recommendations, Assessment, Development and Evaluation
US	United States
HERITAGE	HEalth, RIsk factors, exercise Training And Genetics
RCT	randomized controlled trial
RCTs	randomized controlled trials
PRISMA	Preferred Reporting Items for Systematic Reviews and Meta-Analyses
MCID	minimal clinically important difference
LFK	Luis Furuya-Kanamori
SD	standard deviation
SE	standard error
τ	Tau
$\bar{X}$	mean
Q	Q statistic
*I*^2^	I-squared
Mdn	median
CONSORT	Consolidated Standards of Reporting Trials
Min	minimum
Max	maximum
