# Supplementary material for: Aerobic Exercise Response Variation and Cardiorespiratory Fitness in Adults with Coronary Heart Disease: An SDir Meta-Analysis of Randomized Controlled Trials
Source: J Cardiovasc Dev Dis. 2026 Jul 3;13(7):307. doi: 10.3390/jcdd13070307 (PMC13409841; doi:10.3390/jcdd13070307)
Supplement: Supplementary file 1 [file jcdd-13-00307-s001.zip › IIRD_Exercise_CHD_VO2_JCDD_Supplementary Files.pdf]

List of Supplementary Files

Table S1 – PRISMA Checklist

Table S2 – Study, participant, and intervention characteristics of included studies

Figure S1 – Doi plot for small-study effects (publication bias, etc.) for changes in VO_{2peak} (all studies)

Figure S2 – Forest plot for leave-one-out analysis based on changes in VO_{2peak}

Figure S3 - Forest plot for changes in VO_{2peak} with six outliers deleted from the model

Figure S4 - Doi plot for small-study effects (publication bias, etc.) for changes in VO_{2peak} with six outliers deleted from the model

Table S1. PRISMA Checklist.

Section and Topic	Item #	Checklist item	Location where item is reported (page)
**TITLE**			
Title	1	Identify the report as a systematic review.	1
**ABSTRACT**			
Abstract	2	See the PRISMA 2020 for Abstracts checklist.	1
**INTRODUCTION**			
Rationale	3	Describe the rationale for the review in the context of existing knowledge.	1-3
Objectives	4	Provide an explicit statement of the objective(s) or question(s) the review addresses.	3
**METHODS**			
Eligibility criteria	5	Specify the inclusion and exclusion criteria for the review and how studies were grouped for the syntheses.	4
Information sources	6	Specify all databases, registers, websites, organisations, reference lists and other sources searched or consulted to identify studies. Specify the date when each source was last searched or consulted.	4
Search strategy	7	Present the full search strategies for all databases, registers and websites, including any filters and limits used.	Not applicable
Selection process	8	Specify the methods used to decide whether a study met the inclusion criteria of the review, including how many reviewers screened each record and each report retrieved, whether they worked independently, and if applicable, details of automation tools used in the process.	Not applicable
Data collection process	9	Specify the methods used to collect data from reports, including how many reviewers collected data from each report, whether they worked independently, any processes for obtaining or confirming data from study investigators, and if applicable, details of automation tools used in the process.	4
Data items	10a	List and define all outcomes for which data were sought. Specify whether all results that were compatible with each outcome domain in each study were sought (e.g. for all measures, time points, analyses), and if not, the methods used to decide which results to collect.	4
	10b	List and define all other variables for which data were sought (e.g. participant and intervention characteristics, funding sources). Describe any assumptions made about any missing or unclear information.	4
Study risk of bias assessment	11	Specify the methods used to assess risk of bias in the included studies, including details of the tool(s) used, how many reviewers assessed each study and whether they worked independently, and if applicable, details of automation tools used in the process.	5-6
Effect measures	12	Specify for each outcome the effect measure(s) (e.g. risk ratio, mean difference) used in the synthesis or presentation of results.	5

Section and Topic	Item #	Checklist item	Location where item is reported (page)
Synthesis methods	13a	Describe the processes used to decide which studies were eligible for each synthesis (e.g. tabulating the study intervention characteristics and comparing against the planned groups for each synthesis (item #5)).	4
	13b	Describe any methods required to prepare the data for presentation or synthesis, such as handling of missing summary statistics, or data conversions.	5
	13c	Describe any methods used to tabulate or visually display results of individual studies and syntheses.	5
	13d	Describe any methods used to synthesize results and provide a rationale for the choice(s). If meta-analysis was performed, describe the model(s), method(s) to identify the presence and extent of statistical heterogeneity, and software package(s) used.	5-6
	13e	Describe any methods used to explore possible causes of heterogeneity among study results (e.g. subgroup analysis, meta-regression).	5
	13f	Describe any sensitivity analyses conducted to assess robustness of the synthesized results.	5
Reporting bias assessment	14	Describe any methods used to assess risk of bias due to missing results in a synthesis (arising from reporting biases).	5
Certainty assessment	15	Describe any methods used to assess certainty (or confidence) in the body of evidence for an outcome.	6
**RESULTS**			
Study selection	16a	Describe the results of the search and selection process, from the number of records identified in the search to the number of studies included in the review, ideally using a flow diagram.	6, Flow diagram not applicable
	16b	Cite studies that might appear to meet the inclusion criteria, but which were excluded, and explain why they were excluded.	Not applicable
Study characteristics	17	Cite each included study and present its characteristics.	6-7, Table 1, Table S2
Risk of bias in studies	18	Present assessments of risk of bias for each included study.	5-6
Results of individual studies	19	For all outcomes, present, for each study: (a) summary statistics for each group (where appropriate) and (b) an effect estimate and its precision (e.g. confidence/credible interval), ideally using structured tables or plots.	Table 2, Figure 1, 8-9
Results of syntheses	20a	For each synthesis, briefly summarise the characteristics and risk of bias among contributing studies.	5-6, 8-9
	20b	Present results of all statistical syntheses conducted. If meta-analysis was done, present for each the summary estimate and its precision (e.g. confidence/credible interval) and measures of statistical heterogeneity. If comparing groups, describe the	Table 2, Figure 1, 8-9

Section and Topic	Item #	Checklist item	Location where item is reported (page)
		direction of the effect.	
	20c	Present results of all investigations of possible causes of heterogeneity among study results.	8-9, Table 2, Figure S1, Figure S4
	20d	Present results of all sensitivity analyses conducted to assess the robustness of the synthesized results.	8-9, Figure S2, Figure S3
Reporting biases	21	Present assessments of risk of bias due to missing results (arising from reporting biases) for each synthesis assessed.	5-6, 8-9, Figure S1, Figure S4
Certainty of evidence	22	Present assessments of certainty (or confidence) in the body of evidence for each outcome assessed.	5-6
**DISCUSSION**			
Discussion	23a	Provide a general interpretation of the results in the context of other evidence.	9-10
	23b	Discuss any limitations of the evidence included in the review.	11
	23c	Discuss any limitations of the review processes used.	11
	23d	Discuss implications of the results for practice, policy, and future research.	10-11
**OTHER INFORMATION**			
Registration and protocol	24a	Provide registration information for the review, including register name and registration number, or state that the review was not registered.	4
	24b	Indicate where the review protocol can be accessed, or state that a protocol was not prepared.	4
	24c	Describe and explain any amendments to information provided at registration or in the protocol.	4
Support	25	Describe sources of financial or non-financial support for the review, and the role of the funders or sponsors in the review.	12
Competing interests	26	Declare any competing interests of review authors.	12
Availability of data, code and other materials	27	Report which of the following are publicly available and where they can be found: template data collection forms; data extracted from included studies; data used for all analyses; analytic code; any other materials used in the review.	12

From: Page MJ, McKenzie JE, Bossuyt PM, Boutron I, Hoffmann TC, Mulrow CD, et al. The PRISMA 2020 statement: an updated guideline for reporting systematic reviews. BMJ 2021;372:n71. doi: 10.1136/bmj.n71. This work is licensed under CC BY 4.0. To view a copy of this license, visit https://creativecommons.org/licenses/by/4.0/

Table S2. Study, participant, and intervention characteristics of included studies [41-59,61-63,65–70].

Study and Year	Country	Total N (N CAE, N Control)	Females (%)	Age ($\bar{X}$ ± SD) (CAE, Control)	BMI ($\bar{X}$ ± SD) (CAE, Control)	Intervention (CAE)	VO_2 ($\bar{X}$ ± SD) (CAE, Control)	VO_2 Assessment Modality
Anomasiri & Thongthawee,2005	Thailand	33 (18,15)	nd	58±8.5, 58±7	25.1±3.2, 27.2±3.2	8wks, 4xwk, 30min(treadmill), 50-65%VO_{2peak}, 90% compliance	19.9±6.8, 22.1±7.6	treadmill
Balen et al.,2008	Croatia	60 (30,30)	26.7	59±9, 61±10	28.8±3.8, 28±3.8	3wks, 45min(cycling)/30min(daily walking), 50-60%VO_{2peak}, supervised	20±4.4, 17.9±4.6	nd
Belardinelli et al.,2001	Italy	118 (59,59)	16.1	53±11, 59±10	nd	6wks, 4xwk, 30min(treadmill), 60%VO_{2peak}, supervised, 94% compliance	18.6±4.6, 20.5±4.4	cycle ergometer

Benetti et al.,2010[a]	Brazil	58 (29,29)	0	nd	29.8±4.0, 29.7±4.1	12wks, 5xwk, 45min(aerobic exercises), 75%MHR	32±5.3, 31.5±3.9	treadmill
Blumenthal et al.,2005[b]	United States	86 (44,42)	32.9	62±10.5, 63±9	29.9±5.7, 29.8±4	16wks, 3xwk, 30min(treadmill), 70-85%HRR, supervised, 89% compliance (median)	19.1±6, 20.2±5.2	treadmill
Cardozo et al.,2015 [a]	Brazil	48 (24,24)	29.2	62±12, 64±12	26.8±4.8, 26.9±4.4	16wks, 3xwk, 30min(treadmill), 70-75% MHR, supervised, 75%+compliance	21.8±6, 21.9±6	treadmill
Dressendorfer et al.,1995[b]	United States	50 (38,12)	0	54.8±9.2, 54.8±11.1	nd	5wks, 1-3xwk, 30-35min(treadmill,cycling,arm cranking, rowing), 70%VO_{2max}, 91.3% compliance	19.1±1.9, 19.2±2.8	treadmill

Giallauria et al.,2006	Italy	40 (20,20)	17.5	68.6±2.3, 68.2±2.6	nd	12wks, 3xwk, 30min(cycling), 60%VO_{2peak}, supervised	16.3±1.4, 15.7±1.5	cycle ergometer
Giallauria et al.,2008	Italy	61 (30,31)	27.9	55.9±3.1, 55.1±3.7	25.6±2.4, 25.3±2.9	24wks, 3xwk, 30min(cycling), 66%VO_{2peak}, supervised,100% compliance	16.4±1.6, 16.3±2	cycle ergometer
Giallauria et al.,2011	Italy	75 (37,38)	20.0	61±7, 60±8	27.3±2.2, 28.2±2.8	24wks, 3xwk, 30min(cycling), 68.4%VO_{2peak}, supervised, 86% compliance	16.4±1.5, 16.7±2.2	cycle ergometer
Giallauria et al.,2013	Italy	46 (25,21)	13.0	54±7, 54±9	nd	24wks, 3xwk,30min(cycling), 69%VO_{2peak}, 91% compliance	14±3, 14±5	cycle ergometer

Haennel et al.,1991[c]	Canada	16 (8,8)	0	51.5±11.3, 56.9±4	nd	8wks, 3xwk, 24min(cycling), 70%HRR	21.4±4.81, 18±3.11	cycle ergometer
Hambrecht et al.,2000	Germany	19 (10,9)	0	60±6.3, 61±3	nd	4wks, 6xday, 60min(cycling), 80%VO_{2peak}, supervised	24±4.74, 23.3±3.3	cycle ergometer
Lee et al.,2008	Taiwan	39 (20,19)	0	52±7, 52±9	26.5±3.4, 26.1±3.7	12wks, 3xwk, 20min(cycling), 55-70%VO_{2peak}, supervised	22.2±3.9, 22.7±3.1	cycle ergometer
Lee et al.,2017[d]	South Korea	40 (21,19)	27.5	72.2±4.5, 72.9±4.7	nd	24wks, 3xwk, 30min(treadmill or track walking), 50-65%HRR, supervised	nd	treadmill
Legramante et al.,2007	Italy	82 (43,39)	0	59.6±8.6, 58±7.5	nd	2wks, 12xwk, 30min(cycling), 75-85%MHR, supervised	17.4±2.62, 17.5±2.5	cycle ergometer

Mameletzi et al.,2011	Greece	20 (10,10)	0	71.1±6, 69.1±6.8	nd	28wks, 3xwk, 30min(combined treadmill, cycling, rowing, gym floor exercises), 70%+MHR, supervised, 80%+compliance	21±4.2, 22.2±3.9	treadmill
Mobius-Winkler et al.,2016[a]	Germany	40 (20,20)	24.3	66±5.9, 65.7±7.6	29.4±4.4, 30±4.8	4wks, 6-8xday, 20min, 60% angina-free exercise threshold, supervised, 95% compliance	22.8±4.8, 21.7±5.5	cycle ergometer
Myers et al.,2000	Switzerland	25 (12,13)	0	56±5, 55±7	nd	8wks, 4xwk, 45min(cycling), 83%MHR, supervised	19.4±3, 18.8±3.9	cycle ergometer
Oliveira et al.,2014	Portugal	92 (47,45)	16.3	54.8±10.6, 58.6±10.7	27±3.6, 27.5±3.3	8wks, 3xwk, 30min(cycling), 70-85%MHR, supervised, 78.8% compliance	27.6±7.3, 26.9±5.6	treadmill

Ribeiro et al.,2012	Portugal	38 (20,18)	18.4	54.3±10.8, 57±7.6	28.4±4, 26.6±4.6	8wks, 3xwk, 35min(cycling), 65-75%MHR, supervised, ≥80% compliance	30.8±7.8, 32.6±5.8	treadmill
Santi et al.,2018[a]	Brazil	20 (10,10)	nd	55.1±8.9, 55.1±8.9	nd	12wks, 3xwk, 30min(treadmill), 60-70%MHR, supervised	18.8±3.7, 18.2±4.4	treadmill
Seki et al.,2003	Japan	38 (20,18)	0	69.3±2.9, 70.1±3.7	23.7±2.8, 23.7±3	24wks, 3xwk, 20-30min(various aerobic exercises) at anerobic threshold, supervised and unsupervised	23±3.3, 24.1±5.2	treadmill
Su et al.,2011	Taiwan	29 (17,12)	0	52±9, 52±8	26±3, 26±4	12wks, 3xwk, 20min(cycling), 55-70% VO_{2max}, supervised	22.5±3.7, 21.2±2.1	treadmill
Takeyama et al.,2000	Japan	28 (13,15)	7.1	58.8±6.3, 61.7±8.7	nd	2wks, 2xday, 30min(cycling) at anaerobic threshold, supervised	13.1±1.7, 13.7±2.5	cycle ergometer

Wu et al.,2006[e]	Taiwan	54 (36,18)	0	61.85±7.2, 62.2±9.6	26.2±1.9, 25.2±2.2	12wks, 3xwk, 30-60min(cycling or treadmill for supervised group, fast walking/jogging for home-based group), 60-85% MHR, supervised or unsupervised	16.1±3.7, 16±4.2	cycle ergometer
Yamamoto et al.,1998[f]	Japan	70 (35,35)	nd	60.8±12.2, 63.2±9.9	nd	8wks, 3xwk, 20min(treadmill walking) at anaerobic threshold, supervised	23.2±4.1, 22.5±4.8	treadmill
Zheng et al.,2008	China	57 (27,30)	0	nd	nd	24wks, 3xwk, 30min(cycling) at anaerobic threshold, supervised	12.6±1.5, 11.7±1.9	cycle ergometer

Notes: CAE, continuous aerobic exercise; $\bar{X}$ ± SD, mean ± standard deviation; BMI, body mass, index; VO_2, peak oxygen consumption in $ml \cdot kg^{-1} \cdot min^{-1}$; nd, data not available; wk(s), week(s); min, minutes; MHR, maximum heart rate; HRR, heart rate reserve; [a], exercise group data limited to moderate-intensity group; [b], data for three exercise groups (1,2, or 3 days of training per week) pooled; [c], exercise

group data limited to cycling group; [d,] exercise group data limited to track/walking group; [e,] data for facility and home-based exercise groups pooled; [f,] Data for anterior and inferior myocardial infarction exercise and control groups pooled.

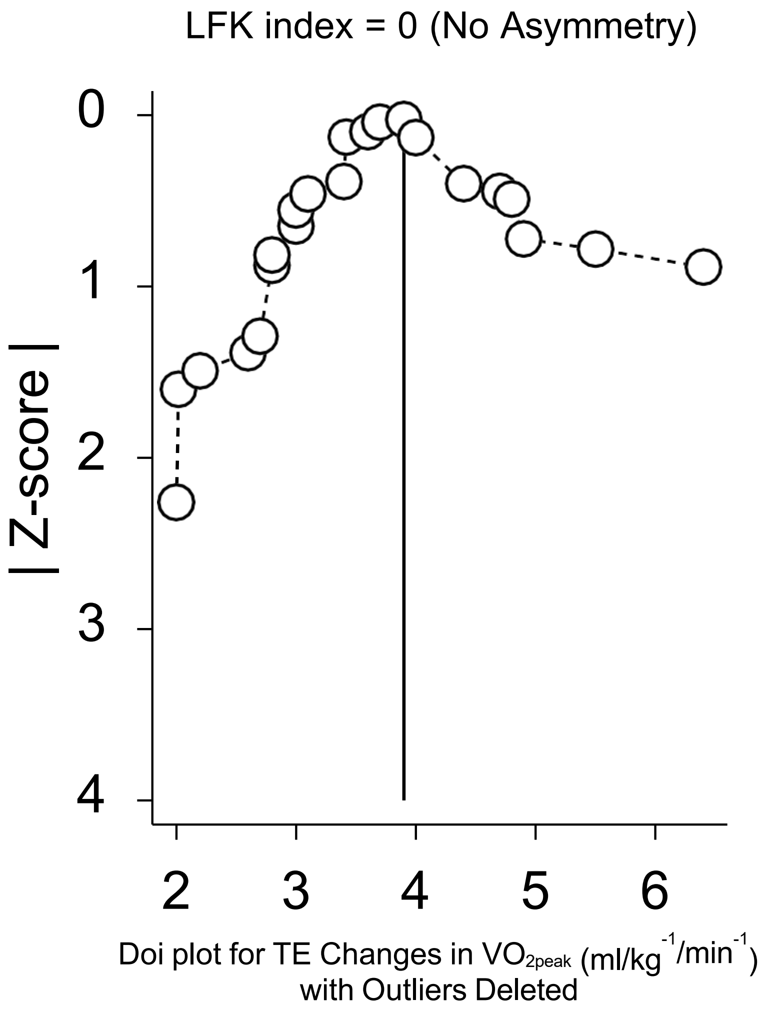

Figure S1. Doi plot for small-study effects (publication bias, etc.) for changes in VO_{2peak} from all studies [41-59,61-63,65–70]. The horizontal (x) axis represents treatment effect changes in VO_{2peak} while the vertical (y) axis represents the Z-score derived from the normal distribution. The solid verrtical line represents the overall treatment effect change in VO_{2peak}.

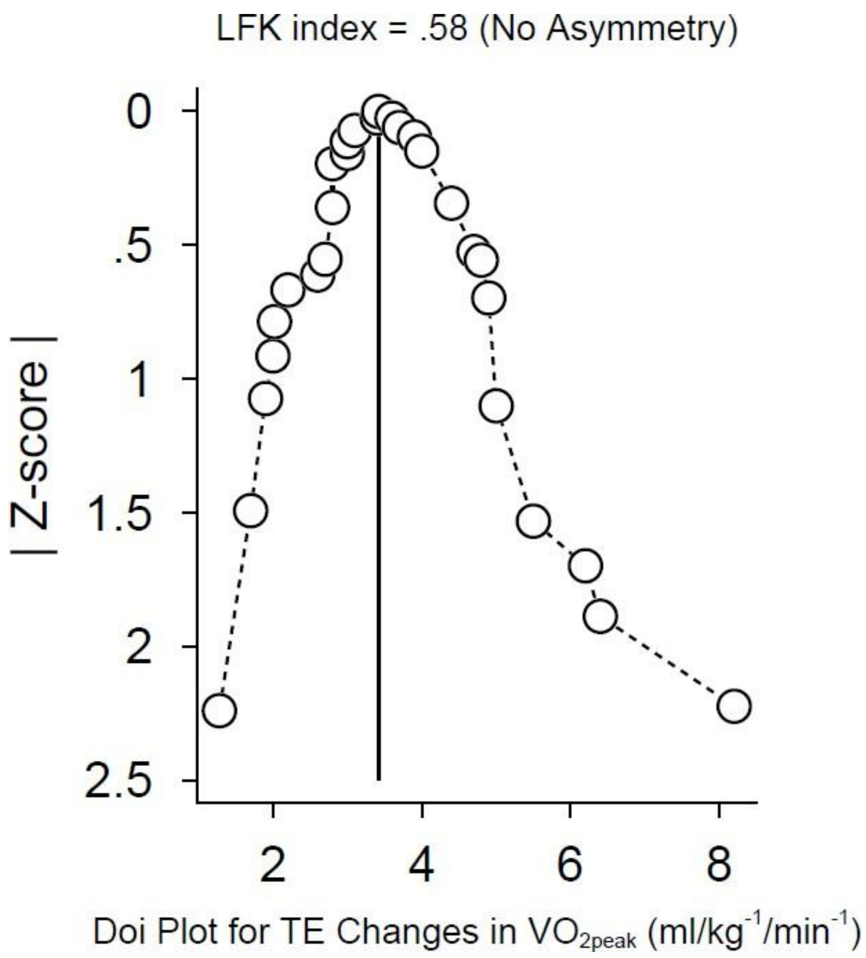
LFK index = .58 (No Asymmetry)
| Z-score |
0
.5
1
1.5
2
2.5
2
4
6
8
Doi Plot for TE Changes in VO2peak (ml/kg-1/min-1)

Figure S2 – Forest plot for leave-one-out analysis based on changes in VO_{2peak}. The left and right sides of the solid horizontal lines represent the lower and upper 95% confidence intervals, the solid (filled) diamond the treatment effect changes in VO_{2peak} and the grey shaded square the total weight with that study deleted from the model. The left and right dashed vertical lines represent the pooled lower and upper 95% confidence intervals with all studies pooled while the dashed vertical line represents the overall pooled treatment effect for all studies. Results ordered from low to high for all studies [41-59,61-63,65–70].

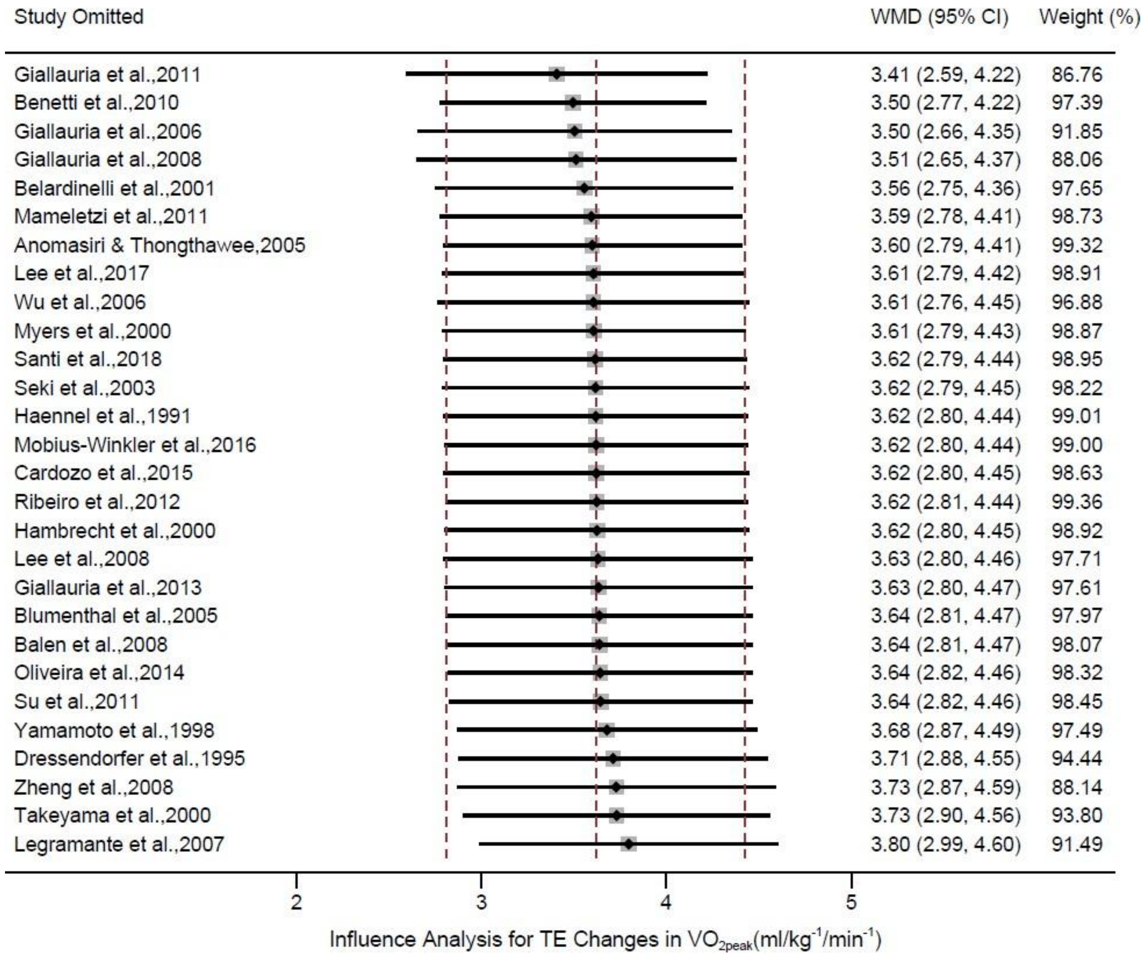

Figure S3 - Forest plot for changes in VO_{2peak} with six outliers deleted from the model [43,44,49,56,67,69]. For each study, the left and right sides of the horizontal lines represent the lower and upper 95% confidence intervals for changes in VO_{2peak}, the solid diamond the mean change in $VO_{2peak,}$ and the grey square the weight applied to that study. The left and right sides of the hollow diamond at the bottom of the figure represent the lower and upper 95% confidence intervals for overal pooled changes, i.e., treatment effect changes, in VO_{2peak}, while the middle of the diamond and dashed vertical line represents the overall mean treatment effect change in $VO_{2peak.}$ Results ordered from low to high for included studies [41,42,45-48,50-55,57-66,68,70].

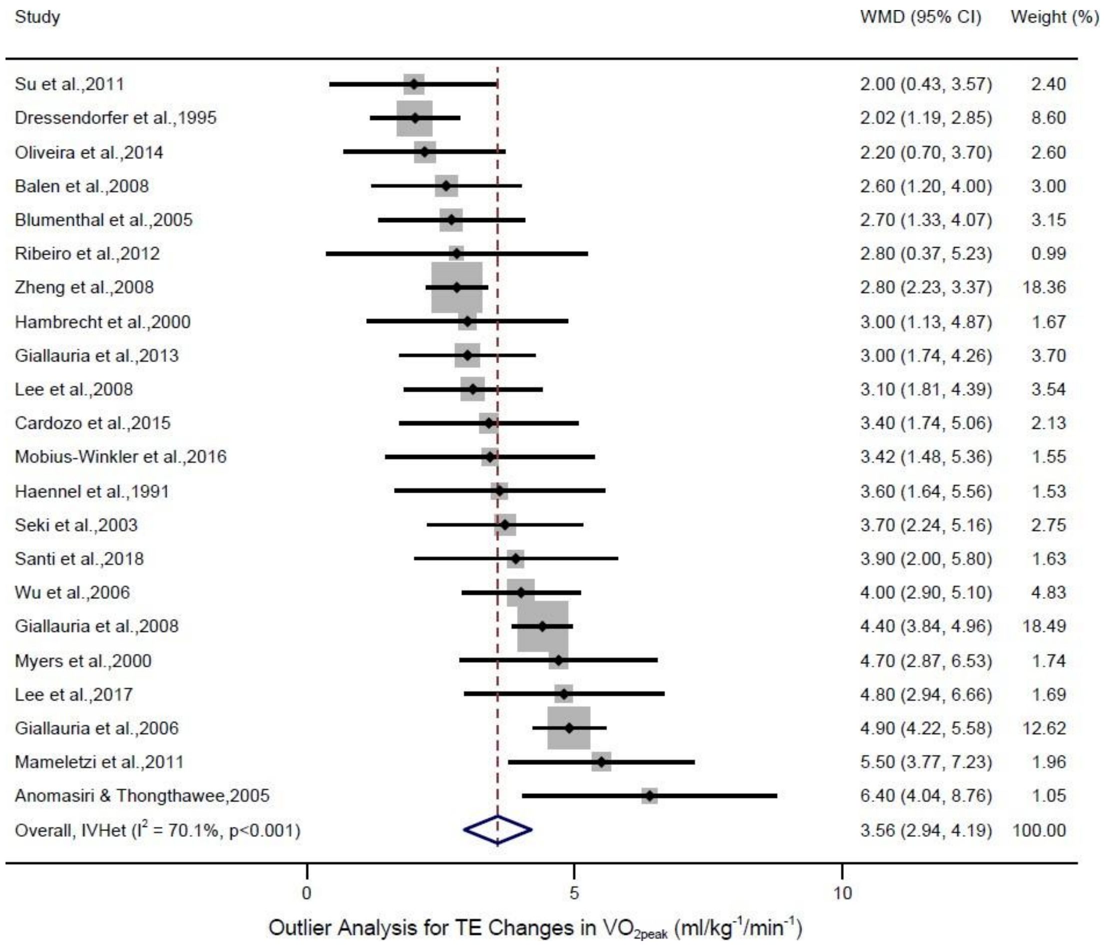

Figure S4 - Doi plot for small-study effects (publication bias, etc.) for changes in VO_{2peak} with six outliers deleted from the model [43,44,49,56,67,69]. The horizontal (x) axis represents treatment effect changes in VO_{2peak} while the vertical (y) axis represents the Z-score derived from the normal distribution. The solid verrtical line represents the overall treatment effect change in VO_{2peak} included studies [41,42,45-48,50-55,57-66,68,70]..
